# HES1 promotes breast cancer stem cells by elevating Slug in triple-negative breast cancer

**DOI:** 10.7150/ijbs.53477

**Published:** 2021-01-01

**Authors:** Xiaoying Li, Yang Li, Xianqiang Du, Xu Wang, Shu Guan, Yu Cao, Feng Jin, Feng Li

**Affiliations:** 1 Department of Cell Biology, Key Laboratory of Cell Biology, National Health Commission of the PRC, and Key Laboratory of Medical Cell Biology, Ministry of Education of the PRC, China Medical University, No. 77, Puhe Road, Shenyang North New Area, 110122 Shenyang, Liaoning, China.; 2 Department of Breast Surgery, The First Affiliated Hospital of China Medical University, 155 Nanjing Road, 110001 Shenyang, China.; 3 Department of Breast Surgery, Affiliated Quanzhou First Hospital of Fujian Medical University, Anji Road, Quanzhou, China.

**Keywords:** stem cells, breast cancer

## Abstract

Triple-negative breast cancer (TNBC) is the most aggressive subtype of breast cancer. TNBC is enriched with breast cancer stem cells (BCSCs), which are responsible for cancer initiation, cancer progression and worse prognosis. Our previous study found that HES1 was overexpressed and promoted invasion in TNBC. However, the role of HES1 in modulating BCSC stemness of TNBC remains unclear. Here, we found that HES1 upregulates Slug both in transcriptional level and in protein level. HES1 also has a positive correlation with Slug expression in 150 TNBC patient samples. TNBC patients with high HES1 and Slug levels show worse prognosis in both progression-free survival and overall survival analyses. Survival analyses indicate that the effects of HES1 on survival prognosis may depend on Slug. Furthermore, we reveal that HES1 is a novel transcriptional activator for Slug through acting directly on its promoter. Meanwhile, HES1 knockdown reduces BCSC self-renewal, BCSC population, and cancer cell proliferation in TNBC, whereas overexpression of Slug restores the oncogenic function of HES1, both *in vitro* and *in vivo*, suggesting that HES1 performs its oncogenic role through upregulating Slug. Taken together, HES1 promotes BCSC stemness properties via targeting Slug, highlighting that HES1 might be a novel candidate for BCSC stemness regulation in TNBC and providing new clues for identifying promising prognostic biomarkers and therapeutic targets of TNBC.

## Introduction

Breast cancer is the most commonly diagnosed malignancy and the leading cause of cancer-associated mortality among women worldwide 1, 2. Triple-negative breast cancer (ER-, PR-, and HER2-negative, TNBC), the most aggressive subtype, accounts for approximately 10-24% of all breast cancers and is associated with advanced stage, high recurrence rate, frequent distant metastasis, chemoresistance and worse prognosis 3. Due to the lack of effective targeted therapy for TNBC, striving for novel biomarkers and specific targets is regarded to be imperative to both theoretical studies and clinical practice.

Recently, more and more research has indicated that TNBC is enriched with cancer stem cells (CSCs), which resulted in remarkably heterogeneous 4. Cancer stem cells (CSCs) represent a dynamic small subpopulation of cancer cells, characterized by self-renewal, pluripotency and limitless proliferative properties 5. Breast cancer stem cells (BCSCs) are considered as the source of cancer aggression and metastasis, poor prognosis, drug resistance and early recurrence in TNBC 6. Therefore, it is essential to identify key stemness regulators and BCSC-related pathways, which can be applied to predict cancer aggressive potentiality and acted as molecular specific targets for the treatment of TNBC.

Hairy and enhancer of split homolog-1 (HES1) is a basic helix-loop-helix (bHLH) transcriptional factor that plays a critical role in many physiological processes and various morphogenetic events, especially gaining and retaining CSC stemness capacity, inhibiting cell committed differentiation 7, 8. HES1 exerts its dual functions of transcriptional activation and repression 9. Recent many studies showed that HES1 overexpression resulted in chemoresistance in lung cancers, intestine cancers, and pancreatic cancers owing to elevating stemness properties acquisition 8, 10, 11. Our previous study also found that HES1 is highly expressed in TNBC than in other subtypes of breast cancer and significantly correlated with advanced TNM stage and poor prognosis. In addition, HES1 overexpression induced epithelial to mesenchymal transition (EMT) process and promoted cancer invasion in TNBC 12. However, it has not been clarified whether HES1 promotes TNBC to gain BCSC phenotype and regulates BCSC stemness properties.

It is well known that EMT is the process characterized by epithelial cancer cells acquiring a mesenchymal gene program which facilitates migration and invasion 13. It has been demonstrated that some transcription factors (such as Twist, Snail, Slug, ZEB1, ZEB2) involved in EMT process, which were counted as EMT-inducing transcription factors (EMT-TFs) 14, 15. Over the past few years, it has been demonstrated that EMT is closely associated with CSC stemness properties 16. In breast cancer, interconversion of CSC and non-CSC might be a relatively common phenomenon that is driven by EMT process 17. Based on the above, it led us to hypothesize that HES1 might be involved in acquisition and maintenance of BCSC stemness properties in TNBC.

In the present study, we investigated the regulatory role of HES1 in EMT-TFs and BCSC stemness in TNBC. First, we found that HES1 could act directly on Slug promoter to activate its transcription, which is a major EMT-TF. Moreover, there were the positively significant correlation between HES1 expression and Slug expression in 150 TNBC patient specimens. Furthermore, HES1 functioned as an important contributor to regulate BCSC stemness and cancer cell proliferation in TNBC through elevating Slug expression, both *in vitro* and *in vivo*. This study might provide theoretical basis for finding novel prognostic markers and therapeutic targets of TNBC.

## Materials and Methods

### Patient specimens

All patients were diagnosed with triple-negative invasive ductal carcinoma in the First Affiliated Hospital of China Medical University. None of the patients had received preoperational radiotherapy or chemotherapy. Breast cancer specimens (n=150 patients) were obtained from patients hospitalized between September 2010 and September 2011. The deadline date of follow-up was September 2020. All 150 patients had a definite histological pathological diagnosis of breast cancer according to the American Joint Committee Cancer (AJCC) standard. The average age of the 150 patients was 47 years. This study was approved by Ethics Committee of China Medical University (Approval number: AF-SOP-07-1.1-01) with written, informed consent was obtained from each participating patient.

### Immunohistochemistry (IHC)

IHC was performed as previously described 12. Briefly, sections from paraffin-embedded tumor tissues from patients underwent surgical dissections or transplanted nude mice were incubated with primary antibodies. Results were evaluated by two pathologists who were blinded to the experiment separately. Slug immunoreactivity was quantified using a combined “H score”, which assesses both the staining intensity and (0, negative; 1, weak; 2, moderate; 3, strong) and the percentage of cells positively stained (0, <5%; 1, 5-25%; 2, 26-50%; 3, 51-75%; 4, 76-100%). HES1 scores were estimated according to previously described 12. Scores of more than or equal to 4 were defined as positive expression.

### Cell culture and transfection

Triple-negative breast cancer cell lines MDA-MB-231 and HCC-1937 were obtained from ATCC (Manassas, VA, USA). Cell lines were cultured in DMEM (Gibco) with 10% FBS (Gibco). MDA-MB-231 CSCs and HCC1937 CSCs were induced and cultured in DMEM-F12 (Gibco, Thermo Fisher Scientific), containing 2% B27 (Gibco), b-FGF 10 μg/L (Promega), EGF 20 μg/L (Promega), as previously described. All cells were incubated in a 5% CO2 air at 37 °C. The shRNA expressing lentivirus for HES1 and the Slug-overexpression lentivirus were purchased from Hanheng Company (Wuhan, China) and GeneChem Company (Shanghai, China). The sequences of HES1-shRNA used to knockdown HES1 were GGACATTCTGGAAATGACA. Puromycin (10 mg/ml; Sigma, USA) was used to select stably transfected cells. Human Slug cDNA was amplified by PCR and cloned into pcDNA3.1 plasmid. Triple-negative cancer cells were transfected with Slug plasmid by Lipofactamine 3000 (Invitrogen, Carlsbad, CA, USA).

### Western blot

In brief, cells were lysed with RIPA buffer containing 1% protease inhibitor cocktail (Roche, Germany) on ice for 30 min and then lysates were centrifuged. Protein concentrations were measured using the BCA assay kit (KeyGen). Cell lysates were separated by SDS-PAGE and transferred to polyvinylidene fluoride membranes (Millipore, Billerica, MA, USA) and the membranes were incubated with primary antibodies overnight at 4 °C. The primary antibodies used in western blot were: anti-HES1 (1:500, ab71559, Abcam), anti-Twist (1:1000, 46702, CST), anti-Snail (1:1000, 3829, CST), anti-Slug (1:1000, 9585, CST), anti-ZEB1 (1:1000, 3396 , CST), anti-ZEB2 (1:1000, 97885, CST), anti-SOX2 (1:1000, ab92494, Abcam), anti-OCT4 (1:1000, 2750, CST), anti-Nanog (1:1000, ab109250, Abcam) and anti-β-actin (1:1000, ab8227, Abcam). All western blots were derived from the same experiment and were processed in parallel.

### RNA isolation and quantitative real-time PCR (qRT-PCR)

Total RNA was extracted from cells using Trizol reagent (Invitrogen, Carlsbad, USA). Quantitative real-time PCR was performed using the SYBR Green mix (Applied Biosystems) on a 7500 Fast Real-Time PCR System (Applied Biosystems). The sequences of the primers were as follows (5′-3′): HES1 (TCAACACGACACCGGATAAAC and GCCGCGAGCTATCTTTCTTCA); Twist (GTCCGCAGTCTTACGAGGAG and GCTTGAGGGTCTGAATCTTGCT); Snail (TCGGAAGCCTAACTACAGCGA and AGATGAGCATTGGCAGCGAG); Slug (CGAACTGGACACACATACAGTG and CTGAGGATCTCTGGTTGTGGT); ZEB1 (GATGATGAATGCGAGTCAGATGC and ACAGCAGTGTCTTGTTGTTGT); ZEB2 (CAAGAGGCGCAAACAAGCC and GGTTGGCAATACCGTCATCC) and GAPDH (GAGTCAACGGATTTGGTCGTATTG and CCTGGAAGATGGTGATGGGATT). The fold change of gene expression levels was quantified using the 2-ΔΔCt method. GAPDH was amplified as endogenous control.

### ChIP (Chromatin immunoprecipitation) assay

Potential transcription factor HES1-binding sequences within Slug promoter region were identified by JASPAR database. According to the protocol of ChIP assay kit (Merk Millipore); MDA-MB-231 cells were cross-linked with 1% formaldehyde at 37 °C for 10 min after washing. Cells were resuspended in lysis buffer, sonicated to shear DNA and sonicates were immunoprecipitated with anti-HES1 antibody (ab70576, Abcam) or negative control IgG at 4 °C overnight. The immunocomplex was collected on protein A/G-agarose and washed in turn with low salt, high salt, lithium chloride buffer and TE buffer. After elution and reverse cross-linking, the DNA was extracted and analyzed by PCR using primer pairs for the Slug promoter: Slug (BE1) forward: 5′-CAAGGAGGACTCCTGCTCTC-3′, reverse: 5′-CCTCTGGCTTTTACTCCAGG-3′; Slug (BE2) forward: 5′-GCTAACACGGTGACATGAGT-3′, reverse: 5′-CACGCAAGGCTGCAGT-3′; Slug (BE3) forward: 5′- CCCTCCTAGCTCCCAGA-3′, reverse: 5′-CCTCTCCACTGAAATCTCAA-3′.

### Luciferase dual reporter assays

The promoter sequence of Slug gene (from -2000 to -1) was obtained from the UCSC website. Partial sequences of the promoter were amplified by PCR, and the fragments were cloned into the pGL3-basic vector. MDA-MB-231 cells were co-transfected with pGL3-basic or pGL3-Slug-Luc together with sh-HES1 or sh-NC, and pRL-TK plasmid. After 24 h, the cells were lysed and luciferase activities were analyzed using a Promega dual-luciferase assay kit according to the manufacturer's instructions.

### Flow cytometry assay

All cell lines were digested with 0.25% trypsin and washed with PBS three times. Then cells were resuspended in 100μL PBS and incubated with anti-CD44-APC (1.25 μl/ test) and anti-CD24-PE (5 μl/ test) (Biolegend, San Diego, USA), or with their controls at 4 °C for 30min. After incubation, the cell distribution was measured using a MACSQuantTM Flow Cytometer (Miltenyi Biotec).

### Sphere-formation assay and colony formation assay

Sphere-formation assay was performed as previously described 18, 19. Essentially, MDA-MB-231 and HCC-1937 cell suspension (1 × 10^3^/well) were plated in ultralow adhesion plates (Corning, Kraemer, CA). The cells grown in 2 ml serum-free DMEM-F12 with 10 μg/L bFGF, 20 μg/L EGF and 2% B27. For Colony formation assay, 2 × 10^3^ MDA-MB-231 and HCC-1937 cells were seeded into 6 cm petri dishes and cultured at 37 °C in 5% CO2 for 14 days. Then, cells were washed with PBS, fixed with 4% paraformaldehyde and stained with 0.5% crystal violet for number counting.

### Xenograft model

Nude female mice (8 weeks-old) were used for studying tumorigenesis ability. Tumorsphese cells with limiting dilution (1.0 × 103; 1.0 × 104; 1.0 × 105 cells per mouse) were injected subcutaneously into second mammary fat pad. We adopted the method of tumorsphere isolation and cells count in our study, which was described in the previous researches in detail 19, 20. In essential, spheres were collected, enzymatically dissociated into single cells by 0.05% trypsin-EDTA solution for 2-3 min at 37 °C. Disaggregate the tumorspheres using a 25 G needle and syringe until a single cell suspension is produced. Single cells were counted by normal cell-counting method or using cell counter (Invitrogen Countess II Automated Cell Counter). The mice were randomized into the following three groups: MDA-MB-231/sh-NC, MDA-MB-231/sh-HES1 and MDA-MB-231/sh-HES1+oe-Slug. The tumor diameter and weights were measured on every 3 days. The tumor volume was calculated by width2 × length/2. All mice were bred at pathogen-free conditions in the Animal Center. All animal experiments were approved by the Institutional Animal Care and Use Committee of China Medical University (Approval number: 2017007 M).

### Statistical analysis

Statistical analyses were conducted using SPSS 20.0 (Chicago, IL, USA) and GraphPad Prism 8.0 software. All data are presented as the means ± standard deviations (SD) and are representative of at least three experiments. Two-sided Student's *t*-test was performed between two groups. The correlation between HES1 and Slug expression was examined by Spearman's rank correlation test. *P*<0.05, *P*<0.01, *P*<0.001 and *P*<0.0001 were considered statistically significant.

## Results

### HES1 upregulates Slug and positively correlates with Slug expression in TNBC

**Figure 1:**
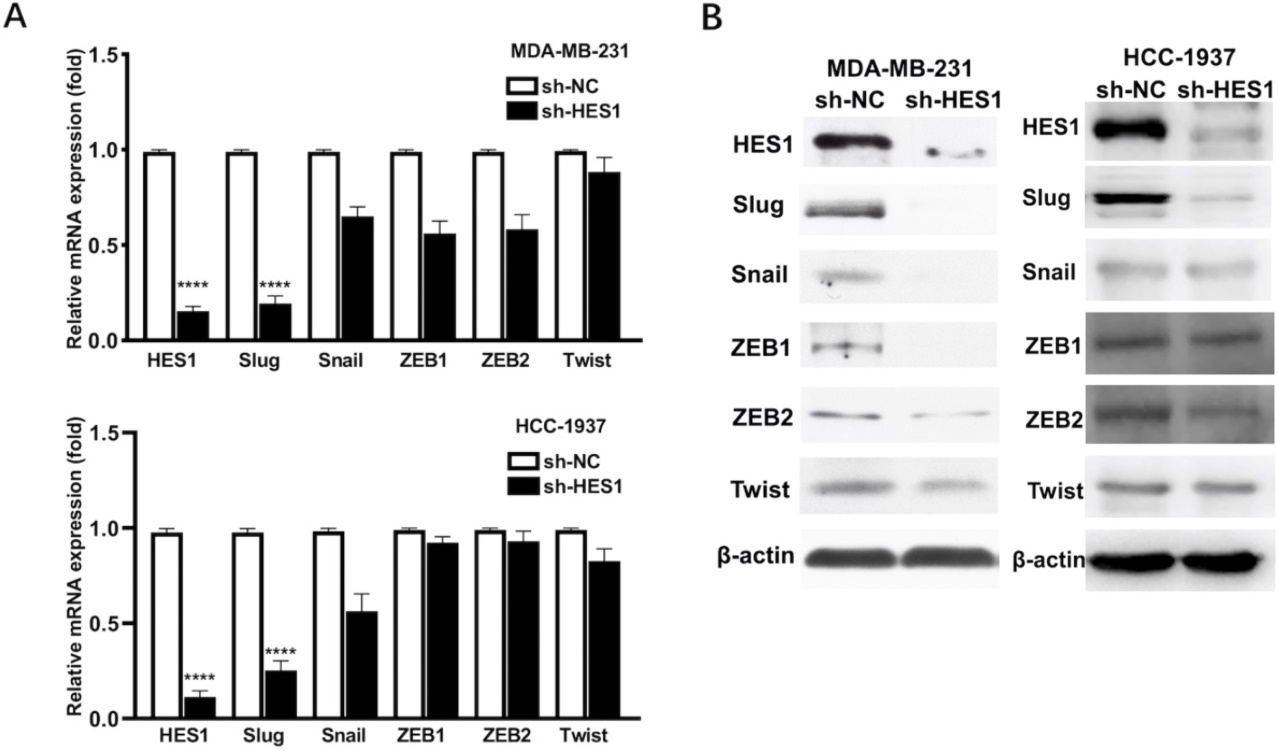
** HES1 upregulates Slug in TNBC.** A) Protein and B) mRNA levels of EMT-inducing transcription factors (Twist, Snail, Slug, ZEB1, ZEB2) was detected in HES1-knockdown TNBC cell lines (MDA-MB-231 and HCC-1937).

**Figure 2:**
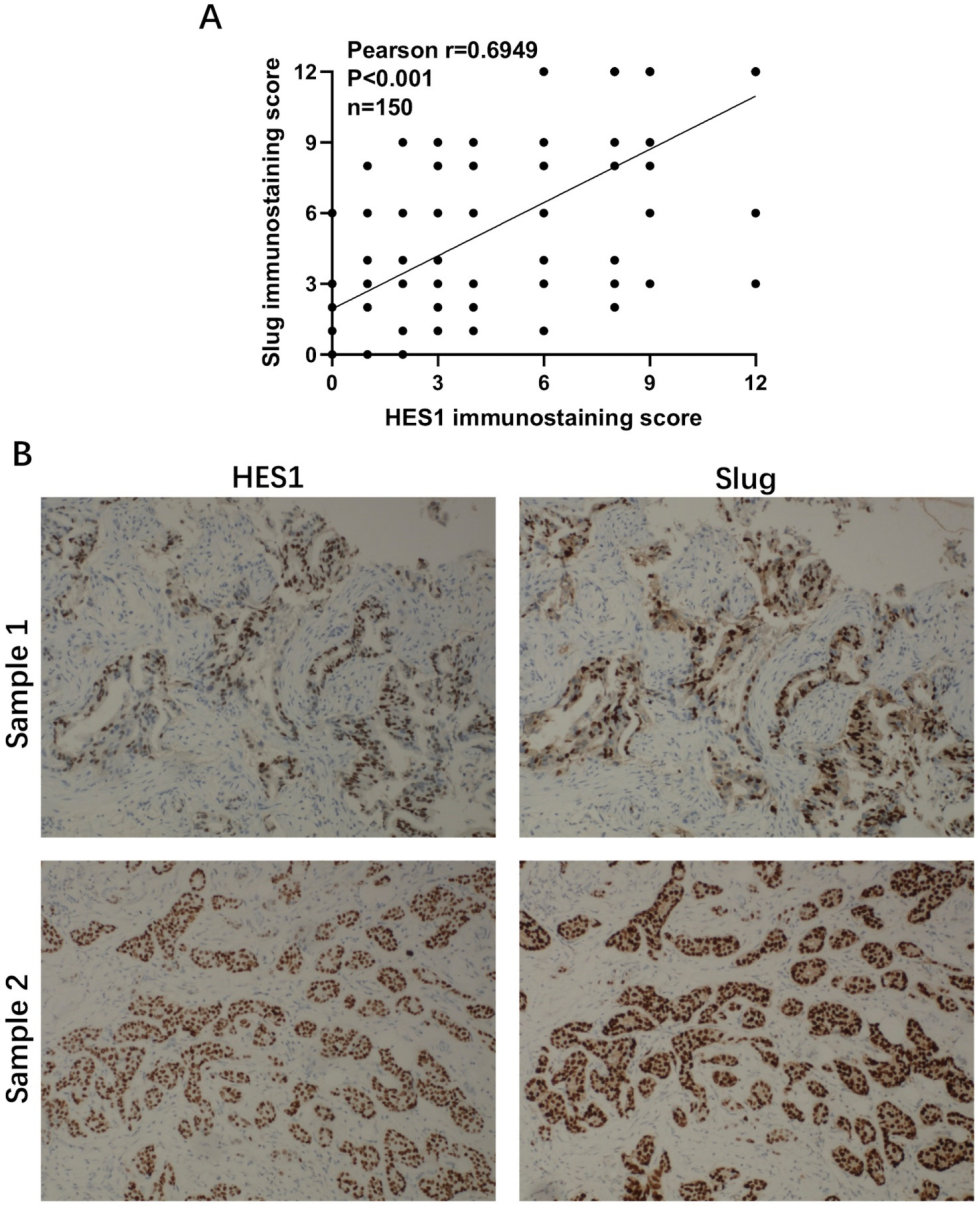
** Correlation analysis of HES1 and Slug in TNBC patients by IHC.** A) The positive correlation between HES1 and Slug expression by IHC scores using Pearson's correlation analysis. B) Representative IHC staining with HES1 and Slug in TNBC tissues.

Our previous study showed that HES1 promotes the EMT process of TNBC cells 12. To further explore how HES1 participates in the activation of EMT process, we examined the expression correlations among HES1 and other EMT-TFs. In TNBC cell lines (MDA-MB-231 and HCC-1937), the mRNA and protein levels of Slug were the most significantly downregulated with loss of HES1 compared with other EMT-TFs (Snail, ZEB1, ZEB2, Twist) (Figure 1). Meanwhile, Slug was high endogenous level in TNBC cell lines, whereas other EMT-TFs (Snail, ZEB1, ZEB2 and Twist) were low endogenous level and slightly expressed in TNBC cell lines (Figure 1). Thus, these data suggested that Slug may serve as a major trigger of EMT in TNBC and HES1 contributes to EMT induction mainly through Slug. Subsequently, in our collected TNBC patient specimens (n=150), there was a significantly positive correlation between HES1 and Slug expression (Figure 2).

**Figure 3:**
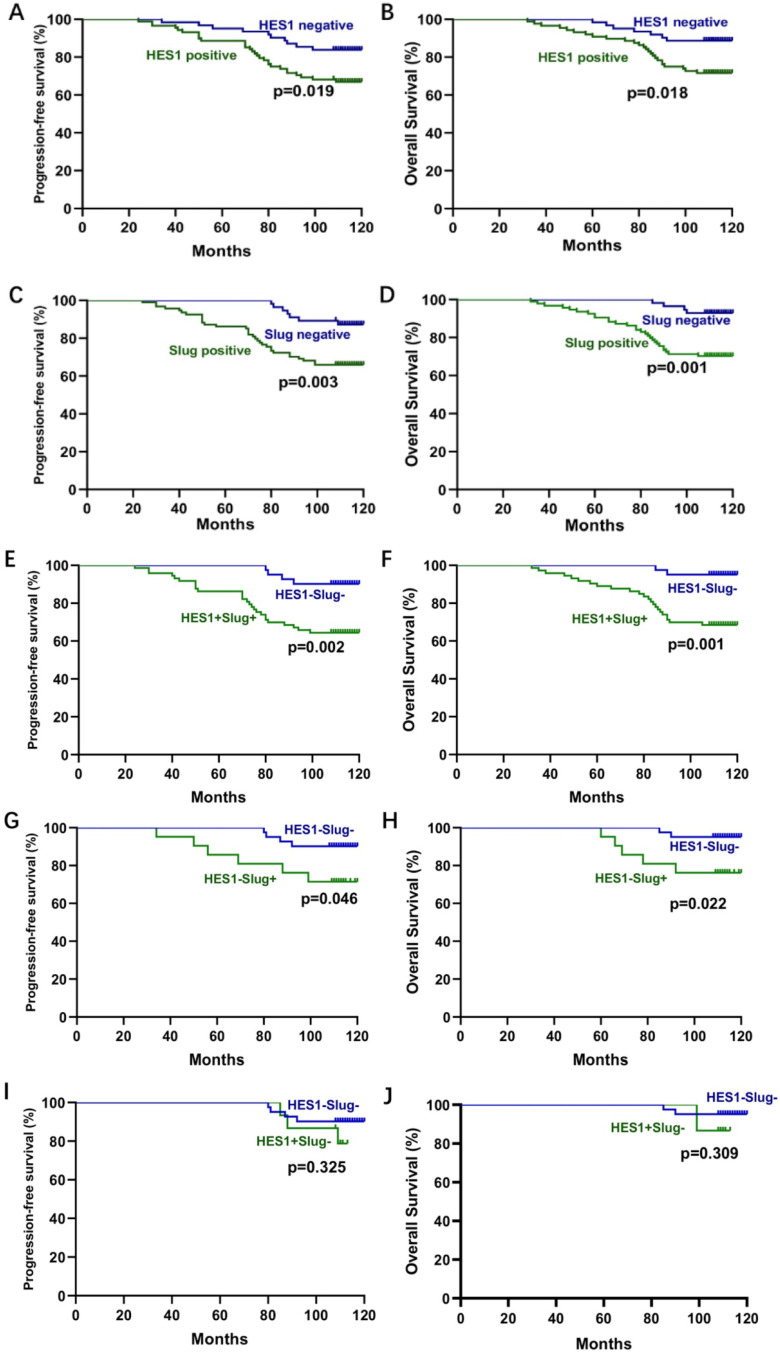
** Correlation analysis between HES1 or Slug expression and survival in TNBC.** A-B) The correlation of HES1 expression with PFS and B) OS of TNBC patients by Kaplan-Meier survival analysis. C-D) The correlation of Slug expression with PFS and OS of TNBC patients by Kaplan-Meier survival analysis. E-J) The correlation of both HES1 and Slug expression with PFS and OS of TNBC patients by Kaplan-Meier survival analysis.

To further evaluate the role of HES1 and Slug in TNBC development, we analyzed the relationship between HES1 and Slug expression and the clinical pathological factors. There was a significant correlation of HES1 or Slug expression with tumor size (*P=*0.0262; *P=*0.0092), lymph node metastasis (*P=*0.0129; *P=*0.0060) and advanced TNM stage (*P=*0.0001; *P=*0.0005) (Table 1). To investigate the prognostic role of HES1 and Slug expression in TNBC, we performed Kaplan-Meier survival analysis. TNBC patients with HES1-positive had a significantly lower progression-free survival (PFS) (*P=*0.019) and overall survival (OS) (*P=*0.018) than TNBC patients with HES1-negative. There were the consistent trends in TNBC patients with Slug-positive (PFS: *P=*0.003; OS: *P=*0.001) compared with TNBC patients with Slug-negative (Figure 3A-D). TNBC patients with both HES1- and Slug- positive significantly correlated with worse prognosis (PFS: *P=*0.002; OS: *P=*0.001) compared with TNBC patients with both HES1- and Slug- negative (Figure 3E-F). In addition, we also analyzed whether Slug level affects survival of TNBC patients in the context of HES1 negative and whether HES1 level has an impact on survival of TNBC patients under the circumstance of Slug negative. As shown in Figure 3G-H, there was a significant relationship between Slug expression and PFS (*P=*0.046) in HES1-negative TNBC patients, as well as there is a remarkable correlation of high Slug level and lower OS (*P=*0.022) in HES1-negative TNBC patients, implying that HES1 may affect survival prognosis dependent on Slug. Meanwhile, as shown in Figure 3I-J, there was no significant association of HES1 expression with PFS (*P=*0.325) or OS (*P=*0.309) in Slug-negative TNBC patients, further indicating that Slug might be a downstream gene of HES1. Thus, the above data suggest that HES1 upregulates Slug in TNBC cells and HES1 level is correlated with Slug level in TNBC specimens. Moreover, HES1 might be an important progressive and prognostic factor in TNBC.

### HES1 promotes Slug transcription

**Figure 4:**
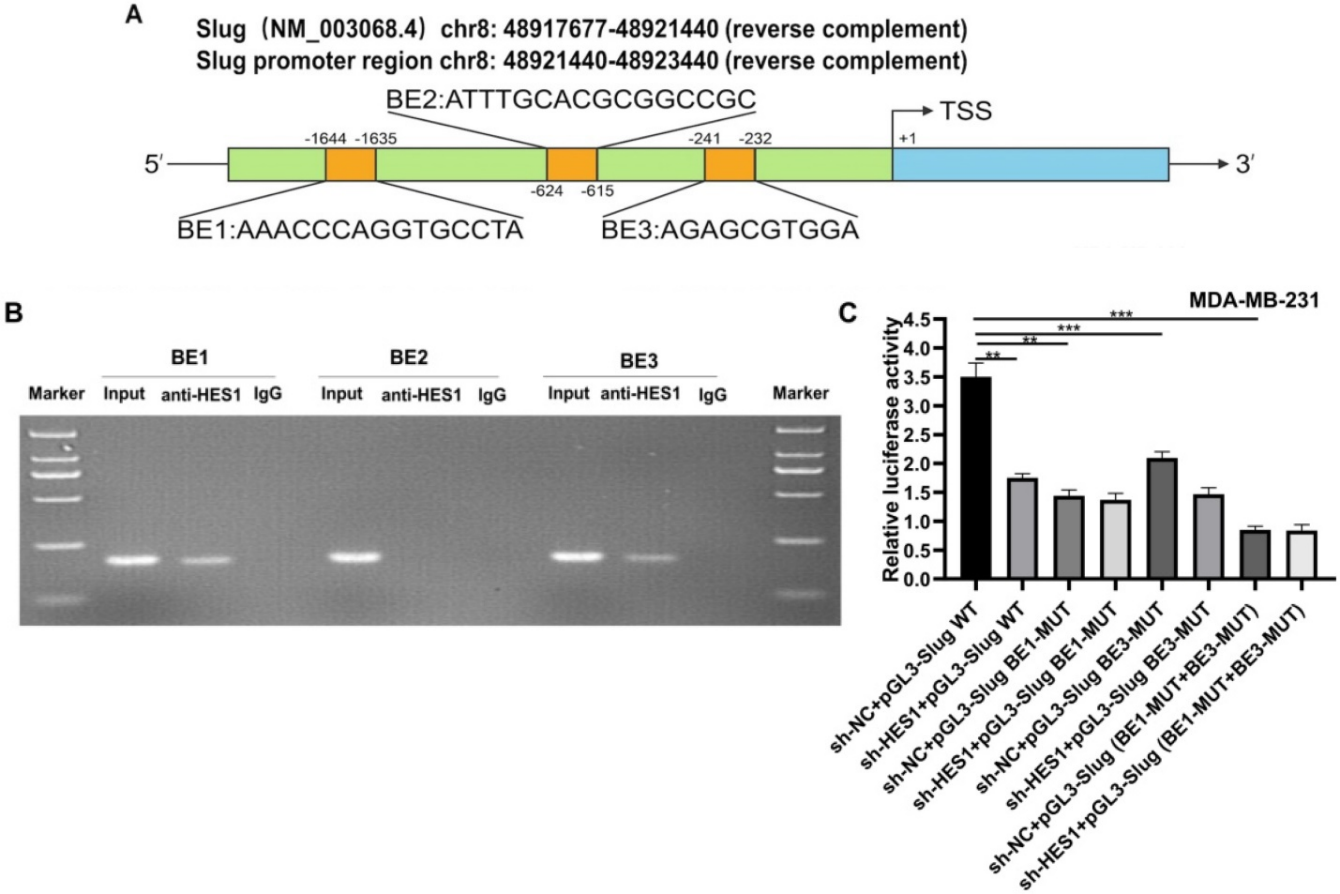
** HES1 activates Slug transcription.** A) The schematic model showed the three putative binding elements (BEs) within Slug promoter region including BE1: 5′-AAACCCAGGTGCCTA-3′ (-1644 to -1635); BE2: 5′-ATTTGCACGCGGCCGC-3′ (-624 to -615); BE3: 5′-AGAGCGTGGA-3′ (-241 to -232). B) HES1-binding sites within Slug promoter was detected in MDA-MB-231 cells by ChIP assay. C) Dual luciferase reporter assay in HES1-knockdown and control MDA-MB-231 cells, with co-transfection of wild-type or mutant-type Slug locus including BE1-MUT, BE1-MUT and BE1-MUT+BE3-MUT. Data are presented as the mean ± SD of three independent experiments performed in triplicate. *P < 0.05, **P < 0.01, ***P < 0.001, ****P < 0.0001.

To investigate whether HES1 functions as a transcriptional activator in regulating Slug expression, we analyzed an approximately 2000bp sequence upstream from the transcriptional start site of Slug gene by bioinformatics analysis. Bioinformatics prediction found that Slug had three putative HES1 binding elements (BEs) including BE1: 5′-AAACCCAGGTGCCTA-3′ (-1644 to -1635); BE2: 5′-ATTTGCACGCGGCCGC-3′ (-624 to -615); BE3: 5′-AGAGCGTGGA-3′ (-241 to -232) (Figure 4A). In order to validate whether HES1 act directly on Slug promoter, we performed ChIP analysis on three putative BEs using HES1 antibody, with IgG as the negative control. The ChIP results revealed that the binding of anti-HES1 antibody was significantly enriched in BE1 and BE3, and there was no enrichment detected with BE2 and IgG control (Figure 4B). To further confirm HES1 binding enhanced Slug transcriptional activity, we conducted dual luciferase gene reporter assay in both MDA-MB-231 cells (sh-NC) and MDA-MB-231 cells with HES1 knockdown (sh-HES1) transfected with Slug promoter wild-type (Slug-WT) and Slug promoter mutant-type (BE1-MUT, BE3-MUT and BE1-MUT+BE3-MUT) luciferase plasmids. The results showed that HES1 knockdown in MDA-MB-231 cells transfected with Slug promoter wild-type plasmid (sh-HES1/Slug-WT) decreased luciferase activity compared with the control group (sh-NC/Slug-WT). Whereas, there was no significant reduction of luciferase activity in HES1-knockdown cells transfected with Slug promoter mutant-type plasmids. Additionally, we also observed that MDA-MB-231 cells transfected with Slug promoter mutant-type plasmids caused a strong decrease in luciferase activity compared with the control group (sh-NC/Slug-WT) (Figure 4C). These results all demonstrated that HES1 promotes Slug gene transcription via binding to Slug promoter region.

### HES1 contributes to BCSC stemness of TNBC through Slug

**Figure 5:**
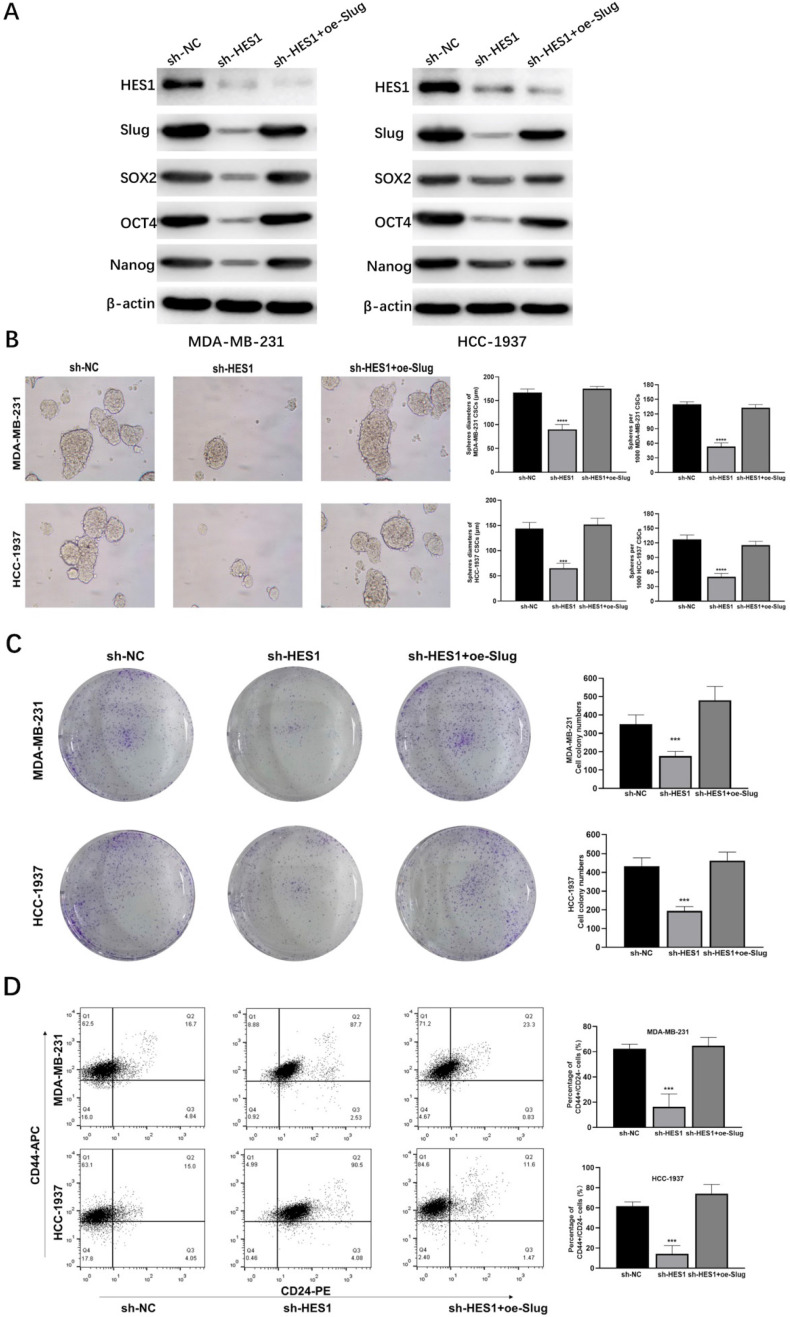
** HES1 regulates BCSC stemness and cancer cell proliferation of TNBC through Slug.** A) Protein levels of stemness-related genes (SOX2, OCT4 and Nanog) was detected in HES1-knockdown and HES1-knockdown with Slug-overexpression TNBC cell lines (MDA-MB-231 and HCC-1937). B) Tumorsphere assay upon HES1 knockdown and HES1 knockdown with Slug-overexpression in TNBC cell lines (MDA-MB-231 and HCC-1937). C) Clonal formation assay upon HES1 knockdown and HES1 knockdown with Slug-overexpression in TNBC cell lines (MDA-MB-231 and HCC-1937). D) Ratio of the CD44^high^CD24^low^ populations was detected in tumorspheres of HES1-knockdown and HES1-knockdown with Slug-overexpression TNBC cell lines (MDA-MB-231 and HCC-1937) by flow cytometry analysis. Data are presented as the mean ± SD of three independent experiments performed in triplicate. *P < 0.05, **P < 0.01, ***P < 0.001, ****P < 0.0001.

To explore whether HES1 is a functional gene in BCSCs and how HES1 maintains stemness of BCSCs in TNBC, we investigated cancer stemness properties by a series of assays and their corresponding rescue assays. In TNBC cell lines (MDA-MB-231 and HCC-1937), the levels of stemness-related proteins such as SOX2, OCT4, Nanog were downregulated with loss of HES1, but restored when Slug was overexpressed upon HES1 knockdown (Figure 5A). HES1 knockdown remarkably reduced the capacity of tumorsphere formation and the ability of clonal formation and in TNBC cell lines (MDA-MB-231 and HCC-1937), but which were restored by upregulating Slug (Figure 5B-C). Based on well-recognized BCSC markers (CD44^high^CD24^low^) 21, BCSC populations (the ratio of CD44^high^CD24^low^ cell fractions) were clearly decreased upon HES1 knockdown in forming tumorspheres of TNBC cell lines (MDA-MB-231 and HCC-1937), whereas also restored by Slug overexpression. (Figure 5D). Thus, the above results all indicate that HES1 contributes to BCSC stemness properties and the impacts of HES1 on BCSC mediated by Slug in TNBC.

### HES1 regulates BCSC stemness of TNBC through Slug *in vivo*

**Figure 6:**
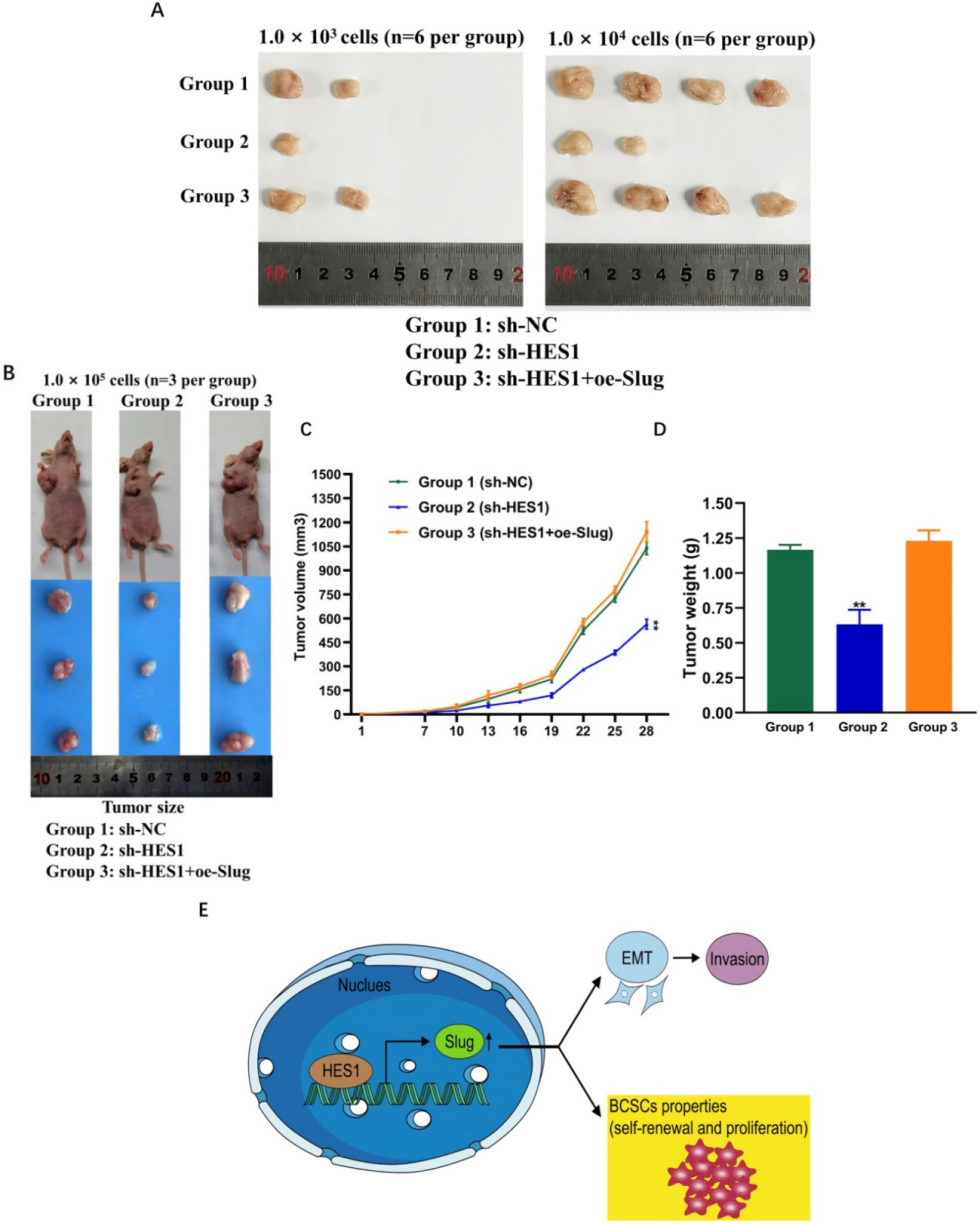
** HES1 regulates BCSC stemness of TNBC through Slug *in vivo*.** A) Xenograft assay using tumorsphere cells (1.0 × 10^3^; 1.0 × 10^4^ cells per mouse, euthanized at seventh week) upon HES1 knockdown and HES1 knockdown with Slug-overexpression in MDA-MB-231 cell line. B) Xenograft assay using tumorsphere cells (1.0 × 10^5^ cells per mouse, euthanized at fifth week) upon HES1 knockdown and HES1 knockdown with Slug-overexpression in MDA-MB-231 cell line. C) Average tumor volumes of mice injected with 1.0 × 10^5^ cells were measured in xenograft mice every three days. D) Average tumor weight of mice injected with 1.0 × 10^5^ cells at the time of dissection. Data are presented as the mean ± SD of three independent experiments performed in triplicate. *P < 0.05, **P < 0.01, ***P < 0.001, ****P < 0.0001. E) The schematic model to show HES1 regulating BCSC stemness of TNBC through Slug as well as HES1 inducing EMT and promoting invasion.

To investigate whether and how HES1 influences BCSC stemness of TNBC *in vivo*, we examined tumor formation probability using mouse xenograft models. We constructed MDA-MB-231 tumorsphere cells mouse xenograft models. There are three groups: group 1: NC mice injected with MDA-MB-231/sh-NC tumorsphere cells; group 2: sh-HES1 mice injected with MDA-MB-231/sh-HES1 tumorsphere cells; group 3: sh-HES1+oe-Slug mice injected with MDA-MB-231/sh-HES1+oe-Slug tumorsphere cells. Consistent with *in vitro* results, transplantations of BCSC populations with limiting dilution assay revealed that the tumor formation probability was severely reduced upon HES1 knockdown and almost rescued by Slug overexpression (Figure 6A, Table 2). HES1 downregulation decreased BCSCs-derived tumor size and weight and the effects were restored by Slug overexpression in xenograft models with the limiting dilution of 1.0 × 105 cells per mouse (Figure 6B-D). The above data suggest that HES1 is closely related with tumor initiation both *in vitro* and *in vivo*. Taken together, our present results combined with our previous data verified that HES1 contributes to BCSC stemness of TNBC through Slug and HES1 upregulated Slug to activate EMT process and promote invasion in TNBC (Figure 6E).

## Discussion

In clinical practice, TNBC possesses the worst prognosis among all subtypes of breast cancers due to the lack of effective specific therapeutic targets. The reason for chemotherapy-resistance and recurrence of TNBC might be BCSCs enrichment in TNBC compared with other subtypes of breast cancers. Because large-scale current therapeutic strategies focus on eliminating the majority non-CSCs, the residual populations are recognized as chemo-resistance cancer cells, which derive from the existence of minority CSCs 22, 23. Thus, seeking for effective therapies aiming at BCSC population of TNBC has attracted considerable attention worldwide. This led us to explore potential specific therapeutic targets for BCSC population of TNBC. Over the past few years, the close connection between CSC and epithelial to mesenchymal transition (EMT) has been demonstrated. The process of EMT not only enhances the migrative and invasive capacities of breast cancer cell, but also endows breast cancer cell with stemness properties 24. Based on these, our previous reported study has demonstrated that HES1 expression is significantly high level in TNBC compared with other subtypes of breast cancer and HES1 leads to EMT process and promotes invasion. In our present study, to further investigate how HES1 regulates EMT, we explored the related mechanism by screening various well-known EMT-TFs including Twist, Snail, Slug, ZEB1, ZEB2 14, 15, both in mRNA and in protein levels. In TNBC cell lines, most of EMT-TFs (Snail, ZEB1, ZEB2, Twist) were low endogenous level and showed mild or insignificant regulatory effects by HES1, but only Slug found to be high endogenously expressed and significantly consistent downregulation by HES1 knockdown. Our findings agree with others, which have demonstrated that Slug is endogenously overexpressed than other EMT-TFs in TNBC cell lines as well as TNBC patients and Slug is markedly higher in TNBC than that for other subtypes of breast cancer 25, 26. The reason for that could be due in large part to the strong correlation between Slug and basal differentiation 25, 27 and also owing to the inverse correlation between Slug and ER expression in both breast cancer tissues and cell lines 28, 29. Basal breast cancer cell almost is the negative for expression of ER, PR and HER2 negative in accordance with characteristics of TNBC by IHC 30, 31. Moreover, Slug (Snai2), as a member of Snail family transcription factors, is well-known to promote cancer progression and cancer-associated EMT in a verity of cancers including breast cancer 32, 33. In TNBC, Slug has been reported to be significantly associated with poor prognosis 25, 26, 28, 34. The above all demonstrated the critical oncogenic role of Slug in breast cancer development.

Thus, we focused on Slug as a critical specific trigger of EMT in TNBC. Meanwhile, we speculated that the remarkably downregulation of Slug by HES1-knockdown may be the main reason for affecting biological functions of TNBC. Subsequently, we revealed that there is a close correlation between HES1 and Slug expression in 150 TNBC patient specimens. Survival analyses indicated that HES1 leads to worse prognosis dependent on Slug. These results provide strong suggestions for that Slug may be a downstream gene of HES1. Moreover, many researches have revealed the critical role of Slug in promoting and maintaining self-renewal capacity of BCSC and inhibiting luminal differentiation. This is consistent with Slug's localization to the basal cell layer of the mammary epithelium, where mammary stem cells reside 25-27, 35, 36, whereas in luminal breast cancer, Snail or ZEB1 but not Slug is predominantly expressed and tightly associated with BCSC self-renewal capacities 25, 37. This provides indications for EMT-driven BCSC stemness properties in different subtypes of breast cancer which is supposed to be triggered by diverse oncogenic signals. We therefore centered on Slug as a specific oncogenic factor responsible for EMT-driven stemness in TNBC, which implied that seeking the upstream regulator of Slug and blocking its regulation will be profoundly important in breast cancer research.

In addition, it has been reported that HES1 plays a critical role in blocking cell differentiation and promoting stemness properties in various cancers such as colon and pancreatic cancers 38, 39. Moreover, several signaling pathways regulate HES1 such as canonical Notch and non-canonical Wnt, Hedgehog, MAPK and TGF-β pathway 30, 41, which all are responsible for CSC stemness properties. Taken together, HES1 might serve as a core factor in the crosstalk of CSC-related signaling pathways 42, 43. And HES1 may become a key biomarker and specific therapeutic target for BCSCs of TNBC. Thus, in our present study, data demonstrated that HES1 is a novel regulatory gene in BCSC self-renewal, BCSC population, cancer cell proliferation, and tumor formation of TNBC.

HES1 functions as a dual transcriptional factor and its role in transcription may depend on the HES1 transcriptional complex 9. When HES1 interacts with a transcriptional co-activator, HES1 promotes its downstream gene transcription. As reported previously, HES1 has been involved in several cancer developments by its role in transcriptional activation or inhibition. In colorectal cancer, HES1 facilitated the aggressive progression via activating Bim1 transcription and repressing PTEN transcription 44. HES1 promoted prostate cancer bone metastasis through an increasing in the transcriptional activity of RUNX2 45, 46. HES1 has been involved in promoting cancer cell transformation and increasing chemoresistance by an enhancement in the STAT3 transcriptional activity 47-49. Considering that Slug is well known to highly correlated with BCSC self-renewal of TNBC 25-27, 35, 36, we speculated that the role of HES1 in regulating BCSCs of TNBC may be mediated by Slug. In our results of ChIP and dual luciferase gene reporter assays, these provided valuable evidences that HES1 is a novel transcriptional activator for Slug gene. Furthermore, the rescue assays from our *in vitro* data on the maintenance of BCSC stemness of TNBC as well as our *in vivo* data on the tumor formation of BCSC of TNBC, indicated that HES1 is involved in stemness of BCSCs in TNBC through Slug. Thus, our findings suggest that necessity of HES1 in the acquisition and maintenance of BCSC stemness of TNBC mediated by Slug.

Our study has some limitations. We selected MDA-MB-231 and HCC-1937 because these cell lines derived from triple-negative breast cancer and expressed endogenous high level of HES1 protein as well as Slug. More cell lines with other molecular subtypes can be used to further prove whether HES1 performs similar function. Although the method of BCSC induction and culture in this research has been approved by many previous reports, BCSCs identification and selection can be improved with more advanced technologies.

In conclusion, based on our previous as well as present findings, we have identified HES1 as a new BCSC maintaining protein for TNBC and found the regulatory network of HES1/Slug/EMT for its oncogenic role in promoting stemness properties in TNBC. Our studies highlighted a mechanism by which HES1 regulates BCSCs stemness properties and induces EMT process in TNBC via acting on Slug promoter region and activating its transcription. We propose that HES1 could serve as a crucial prognostic biomarker of TNBC and targeting HES1 might be a specific effective therapeutic strategy for TNBC in clinical practice.

## Figures and Tables

**Table 1:** Association of HES1 and Slug expression with the clinical pathological characteristics in TNBC

Factors	Number	HES1 expression			Slug expression		
**Age**		Positive	Negative	*P*-value	Positive	Negative	*P*-value
≤47	69	40	29	0.7539	42	27	0.7923
>47	81	49	32		51	30	
**Tumor size (cm)**							
≤3	77	39	38	0.0262*	40	37	0.0092*
>3	73	50	23		53	20	
**LN metastasis**							
Negative	63	30	33	0.0129*	31	32	0.0060*
Positive	87	59	28		62	25	
**TNM stage**							
I	37	12	25	0.0001*	14	23	0.0005*
II-III	113	77	36		79	34	

*Indicated statistical significance (*P* < 0.05).

**Table 2:** Statistical results of xenograft assay using tumorsphere cells upon HES1-knockdown and HES1-knockdown with Slug overexpression in TNBC cell line

Cell number	sh-NC	sh-HES1	sh-HES1+oe-Slug
1000 cells	2/6	1/6	2/6
10000 cells	4/6	2/6	4/6
